# Stress and Corticosteroids Aggravate Morphological Changes in the Dentate Gyrus after Early-Life Experimental Febrile Seizures in Mice

**DOI:** 10.3389/fendo.2018.00003

**Published:** 2018-01-26

**Authors:** Jolien S. van Campen, Ellen V. S. Hessel, Kirsten Bohmbach, Giorgio Rizzi, Paul J. Lucassen, Sada Lakshmi Turimella, Eduardo H. L. Umeoka, Gideon F. Meerhoff, Kees P. J. Braun, Pierre N. E. de Graan, Marian Joëls

**Affiliations:** ^1^Department of Translational Neuroscience, Brain Center Rudolf Magnus, University Medical Center Utrecht, Utrecht, Netherlands; ^2^Department of Child Neurology, Brain Center Rudolf Magnus, University Medical Center Utrecht, Utrecht, Netherlands; ^3^Swammerdam Institute for Life Sciences, Center for Neuroscience, University of Amsterdam, Amsterdam, Netherlands; ^4^Neursocience and Behavioral Sciences Department, Ribeirão Preto School of Medicine, University of São Paulo, Ribeirão Preto, Brazil

**Keywords:** stress, corticosteroids, epilepsy, epileptogenesis, febrile seizures, hyperthermia, early-life

## Abstract

Stress is the most frequently self-reported seizure precipitant in patients with epilepsy. Moreover, a relation between ear stress and epilepsy has been suggested. Although ear stress and stress hormones are known to influence seizure threshold in rodents, effects on the development of epilepsy (epileptogenesis) are still unclear. Therefore, we studied the consequences of ear corticosteroid exposure for epileptogenesis, under highly controlled conditions in an animal model. Experimental febrile seizures (eFS) were elicited in 10-day-old mice by warm-air induced hyperthermia, while a control group was exposed to a normothermic condition. In the following 2 weeks, mice received either seven corticosterone or vehicle injections or were left undisturbed. Specific measures indicative for epileptogenesis were examined at 25 days of age and compared with vehicle injected or untreated mice. We examined structural [neurogenesis, dendritic morphology, and mossy fiber sprouting (MFS)] and functional (glutamatergic postsynaptic currents and long-term potentiation) plasticity in the dentate gyrus (DG). We found that differences in DG morphology induced by eFS were aggravated by repetitive (mildly stressful) vehicle injections and corticosterone exposure. In the injected groups, eFS were associated with decreases in neurogenesis, and increases in cell proliferation, dendritic length, and spine density. No group differences were found in MFS. Despite these changes in DG morphology, no effects of eFS were found on functional plasticity. We conclude that corticosterone exposure during early epileptogenesis elicited by eFS aggravates morphological, but not functional, changes in the DG, which partly supports the hypothesis that ear stress stimulates epileptogenesis.

## Introduction

Epilepsy is a common neurological disorder, especially in childhood where its prevalence is as high as 0.5–1.0% (1). An important factor influencing epilepsy and epileptic seizures is stress, which is the most frequently self-reported seizure precipitant in patients with epilepsy [reviewed in Ref. (2)]. The seizure precipitating effects of stress are also confirmed by prospective studies (3–7). Besides direct effects on seizure susceptibility, stress can also influence the risk of being diagnosed with epilepsy later in life (8–10). Thus, associations between stress and epilepsy exist on multiple levels. However, the mechanisms behind these relations are so far poorly understood.

Animal models can provide more insight into the exact mechanisms by which stress influences epilepsy. In various preclinical epilepsy models, stress has been shown to lower the threshold for the induction of epileptic seizures and to increase seizure severity (11–16). The effects of stress on epilepsy are largely attributed to neuronal exposure to stress hormones. Especially stress hormone exposure early in life can have profound effects on later brain morphology and function and predispose to the development of brain diseases [as reviewed in Ref. (17–21)]. Stress hormones have been shown to directly affect neuronal excitability [reviewed in Ref. (22, 23)]. Despite these effects of stress on seizures on the one hand, and on brain development on the other, effects of stress and stress hormones on the *development* of epilepsy (i.e., epileptogenesis) are currently unknown.

To improve insight into the impact of stress hormones on epileptogenesis, we studied the effects of corticosterone, an important stress hormone, during early-life epileptogenesis on neuronal morphology and functional plasticity in the rodent brain. Using a controlled design, we elicited experimental febrile seizures (eFS) in young mouse pups by warm-air induced hyperthermia (HT) and subsequently exposed them to repetitive (1) high concentrations of corticosterone, (2) vehicle injections (a control condition that is also a mild stressor), or (3) no injections. We next examined alterations in morphological and functional parameters in the dentate gyrus (DG), a hippocampal subarea that is affected by eFS (24–28) as well as stress hormones (29). To assess morphological changes, we investigated neurogenesis, cell proliferation, dendritic morphology, spine density, and mossy fiber sprouting (MFS). Functional plasticity was assessed measuring glutamatergic transmission and long-term potentiation (LTP) in the DG. We hypothesize that corticosterone aggravates the epileptogenic changes after eFS.

## Materials and Methods

### Animals

Breeding pairs of C57BL6/J mice were obtained from The Jackson Laboratory (Bar Harbor, ME, USA) and subsequently bred in-house. Litters used in this experiment were derived from multiple breeding pairs (*n* _=_ 99). On postnatal day (P) 1, litters were culled to four to six pups consisting of both males and females. The pups were not weaned during the experiment. Animals were kept in a controlled 12-h light–dark cycle (light on 7 a.m. to 7 p.m.) with a temperature of 22 ± 1°C. Food and water were available *ad libitum* (2111 RMH-TM diet; Hope Farms, Woerden, the Netherlands). All animals were housed in transparent Plexiglas cages (Macrolon type II) with sawdust bedding and paper tissues for nest building. Cages were cleaned at P7 and at P17/18 (in between injection days) by replacing half of the sawdust bedding. All experimental procedures were performed according to the institutional guidelines of the University Medical Center Utrecht and approved by the committee on ethical considerations in animal experiments of Utrecht University (DEC Utrecht, permit number 2012.I.03.047). All efforts were made to minimize suffering of the animals. Pups were assigned to multiple treatment groups per litter. A maximum of two pups per litter was used per treatment group per method of analysis to minimize litter effects on outcome measures. All animal experiments were performed within a period of 6 months and animals in all treatment groups were tested across the whole period to control for environmental or seasonal variation. For the purpose of this study, experiments were only performed on male animals.

### Corticosterone Levels after Injection

To evaluate corticosterone levels after injection with corticosterone or vehicle, naïve P12 mice (*n* = 4–6 per time point per treatment group) were injected intraperitoneally with corticosterone (corticosterone-HBC complex 3 mg/kg dissolved in saline, total injection volume of 10 μl/g body weight) or vehicle (both obtained from Sigma-Aldrich, the Netherlands) between 8.30 and 9.00 a.m. Immediately before, or at 15, 30, 60, 120, 180, or 240 min after injection, mice were decapitated and trunk blood was collected. Between injection and decapitation, pups were returned to their home cage and left undisturbed. A separate group of non-injected mice was decapitated at the same time points to control for diurnal corticosterone variability.

### Epileptogenesis

Epileptogenesis was induced using the HT-induced eFS model, a very subtle epilepsy model with close resemblance to the human situation in which children who experience complex febrile seizures are at increased risk to develop temporal lobe epilepsy later in life (30, 31). A unique aspect of this model is the relatively long-lasting latent phase of epileptogenesis, making it easier to study effects of additional risk factors prior to the actual onset of epilepsy, and irrespective of the damage and compensatory mechanisms induced by spontaneous seizures.

Prolonged eFS were induced in P10/11 mice by heated-air induced HT using a previously described paradigm in rats (32), which we adapted to mice ((33)). A temperature-sensitive transponder (IPTT-300 BioMedic Data Systems, Plexx BV, Elst, the Netherlands) was implanted subcutaneously on P9/10 in pups with a bodyweight between 5.0 and 6.5 g. One day after transponder implantation, body weight was determined and mice were placed in a preheated cylindrical chamber and exposed to a warm air stream of 41–48°C. To prevent skin burn and adverse effects on behavior, the temperature of the chamber floor was maintained at 39°C. Core body temperature was measured at least every 2.5 min period using a wireless temperature reader (WRS-6007; Plexx BV). To provoke prolonged seizures, air temperature was adjusted to maintain the core body temperature between 41.5 and 42°C. The presence of tonic–clonic convulsions was monitored by observation. These behavioral seizures correlate closely with electroencephalographic seizures, i.e., spike-wave discharges in the hippocampus, as shown by previous experiments in our lab (27, 34). After 30 min of HT (defined as core temperature ≥ 39°C) pups were rapidly cooled in a water bath at room temperature, gently dried with paper and returned to the dam. This procedure is known to induce epileptogenesis, as spontaneous (encephalographic) seizures are observed after a latent period of approximately 3 months in 35–68% of animals (26, 35, 36), and epileptiform interictal discharges in 88% (35). normothermia (NT) controls were treated as HT pups, except that the temperature of the air stream was kept at 30–32°C, resulting in a constant body temperature. All eFS experiments were performed between 10.00 a.m. and 3.00 p.m.

### Injections and Decapitation

**Figure 1:**
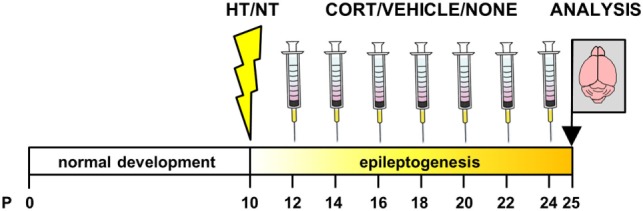
Experimental design. Animals were exposed to hyperthermia (HT) or normothermia (NT) at postnatal day (P) 10 or 11 and subsequently received injections with corticosterone, vehicle or no injection every other day in the latent phase of epileptogenesis. Brains were dissected 24 h after the last injection, before the onset of spontaneous seizure activity.

Animals were weighed at 2, 4, 6, 8, 10, 12, and 14 days after exposure to HT/NT and injected intraperitoneally with corticosterone or vehicle between 8.00 and 9.30 a.m., during the circadian trough. A separate group of animals was left undisturbed after HT/NT. One day (~24 h) after the last injection, mice were weighed and decapitated or perfused (see Figure 1). Decapitation was performed between 8.00 and 9.30 a.m.

### Endocrinology

Trunk blood samples were collected immediately after decapitation at P25/26 from all animals subjected to HT/NT except those receiving perfusion fixation. Directly after decapitation, thymus and both adrenals were resected and weighed. Blood samples were centrifuged for 10 min at 4,000 rpm at 4°C. Plasma was stored at −80°C until assayed with an I^125^-corticosterone radioimmunoassay for mice (MP Biomedicals, Inc., Aberdeen, UK) according to the manufacturer’s instructions. All samples were processed in the same assay to exclude inter-assay variability.

### Morphology and Functional Plasticity

All morphological and functional outcome parameters were examined in the DG. This hippocampal area was selected based on its functional and anatomical characteristics. First, the (epileptogenic) changes induced by eFS are located in the hippocampus, including the DG (24–28, 36, 37). Second, the DG has an important function in filtering excitation and seizure propagation to the other parts of the hippocampus (38, 39). Third, it is one of the few sites where neurogenesis continues in later life, a process that can be stimulated by seizures and has been implicated in epileptogenesis (40–44). Finally, the DG exhibits abundant receptors for corticosterone and therefore its morphology and function are largely influenced by early-life stress (29).

All following experimental procedures were performed by experimenters unaware of the treatment groups. Per outcome measure, tissue of animals belonging to different treatment groups was ordered in a semi-randomized way to control for environmental or experimenter effects on recording, staining and/or quantification over the total experimental period.

#### Neurogenesis

Male mice (*n* = 6 per group) were decapitated between 9.00 and 10.30 a.m. Brains were dissected, post fixed at 4°C in 4% formaldehyde for 4 h, transferred to 30% sucrose for 24 h, frozen on powdered dry ice and stored at −80°C. Cryostat sections (20 μm) were cut in the coronal plane, collected in series of 15 on Superfrost slides and stored at −80°C until further use.

As a measure of neurogenesis, tissue was stained for Doublecortin (DCX), a marker for neuronal precursor cells and immature neurons, and Ki67, a marker for proliferating cells using modified protocols. Mounted sections were postfixed in acetone-methanol (1:1) at −20°C for 7.5 min, washed in 0.05 M Tris HCl 0.9% saline (TBS) pH 7.6 and heated in 0.01 M citrate buffer (pH 6.0) in a microwave oven for 10 min at 800 W followed by 5 min at 480 W and 5 min at 260 W. After a cool down period of 20 min, sections were washed in TBS. Endogenous peroxidase activity was blocked with 0.5% (for DCX) or 1.5% (for Ki67) H_2_O_2_ in TBS for 15 min. Sections were washed and, after incubation in 2% milk powder in TBS for 30 min, incubated with the primary antibody [DCX (polyclonal goat anti-DCX, SantaCruz; 1:800) or Ki67 (polyclonal rabbit anti-Ki67p, Novocastra, 1:5,000)], diluted in supermix (0.25% gelatine and 0.1% Triton in TBS) at room temperature for 1 h and then incubated overnight at 4°C. The next morning, sections were washed and incubated for 2 h with donkey antigoat biotinylated (for DCX, Jackson; 1:500) or goat antirabbit biotinylated (for Ki67, Vector; 1:200) secondary antibody diluted in supermix at room temperature. Sections were washed, incubated in avidin–biotin complex (ABC) (ABC Elite, Vector Laboratories; 1:800 in TBS) for 2 h (for DCX) or 1.5 h (for Ki67) at room temperature, washed again and incubated with biotinylated tyramide (1:500) in 0.01% H_2_O_2_ in TBS for 30 min. Sections were washed and incubated in ABC (1:800 in TBS) for 1.5 h at room temperature and washed in TBS. After washing in 0.05 M Tris HCl pH 7.6 (TB), chromogen development was performed with diaminobenzidine (DAB; 50 mg/100 ml Tris buffer, pH 7.6, 0.01% H_2_O_2_, 0.05% Nickel) for 40 min. Sections were washed in TB and stored overnight at 4°C. The next day, sections were washed in distilled water, counterstained with Hematoxylin and shortly rinsed in distilled water. After washing with running tap water, sections were dehydrated using a grading series of ethanol, cleared in xylene and coverslipped using Entallan.

DCX^+^ and Ki67^+^ cells in the granule cell layer and the subgranular zone of the DG were quantified unilaterally in every 15^th^ section in a total of five sections per animal within Bregma range −1.46 to −2.80 (coronal) without a left/right preference within or between animals. DCX^+^ cells were quantified stereologically using a StereoInvestigator system (Microbright field, USA) with a ×100 oil-immersion objective of a Zeiss Axiophot microscope and StereoInvestigator software, according to the optical fractionator method. The number of DCX^+^ cells was estimated using a 25 μm × 25 μm counting frame, with a grid size of 70 μm × 80 μm. Section thickness was 10.5 μm. The estimated total of DCX^+^ cells within the studied range was determined using the optical fractionator method and multiplied by two to correct for unilateral counting. The mean Gundersen coefficient of error of stereological quantification (*m* = 1) was 0.076 (range 0.06–0.10). Ki67^+^ cells were counted manually using a light microscope (Olympus BH-2) with ×40 magnification and multiplied by the inverse of the sampling fraction and by two to correct for unilateral counting. The total estimated number of DCX^+^ or Ki67^+^ cells per animal was used for analysis.

#### Dendritic Morphology

Male mice (*n* = 6 per group) were decapitated between 9.00 and 10.30 a.m. Directly after decapitation, brains were dissected. Rapid Golgi staining (FD rapid-Golgi staining, Neurotechnologies) was performed according to the manufacturer’s instructions with an impregnation time of 9 days. Vibratome sections (200 μm) were cut in the transversal plane (Leica VT 1000S; Leica Microsystems, Nussloch, Germany). Images were obtained using Zen 2011 (Carl Zeiss) in combination with an automated stage and focus control connected to the microscope. Golgi-impregnated dentate granule cells, fulfilling the following criteria, were randomly selected: (1) localization in the middle part (relative to the DG curvature and start of the CA3 region) of the suprapyramidal blade of the DG, within Bregma range −2.16 and −3.16 (transversal), (2) consistent and dark impregnation along the entire extent for all dendrites, and (3) relative isolation from neighboring impregnated neurons to avoid interference with analysis.

For morphological quantification, eight neurons from each animal in each treatment group were traced. Image stacks of 0.5 μm thickness were automatically acquired and combined. Neurons were traced using NeuroLucida software (MicroBrightField, Inc., Colchester, VT, USA) to obtain a 3D representation of each cell. Numerical analysis and graphical processing were performed with NeuroExplorer (MicroBrightField). Traced dendritic trees were evaluated by two investigators unaware of the treatment, on completeness of staining/tracing and on whether they belonged to a single cell, followed (if required) by a consensus meeting. Dendritic trees that were considered not completely stained/traced or belonging to multiple neurons were excluded from analysis [*n* = 50 (17%), 5–10 neurons per group, evenly distributed over the groups]. Spines were counted in two segments of ±20 μm per neuron, located on different dendrites. Segments were randomly chosen based on the following criteria: (1) localization at approximately 100 μm (80–120 μm) radial distance from the cell soma; (2) secondary or higher order dendritic branches; and (3) straight and remaining in a single focal-plane. Of each cell, dendritic properties were evaluated by analyzing the total dendritic length and maximum dendritic reach (radial distance), and the dendritic complexity index [(Σ branch tip orders + # branch tips) × (total dendritic length/total number of primary dendrites) (45)]. If dendritic length significantly differed between treatment groups, *post hoc* Sholl plots (46) were constructed by plotting the dendritic length as a function of radial distance from the soma center in 18 μm intervals. To normalize the distribution of the dendritic complexity index, neurons with a dendritic complexity index > 2 SD from the group mean were considered outliers and removed from the respective analysis [*n* = 12 (5%), 1–3 neurons per group, evenly distributed over the groups]. The remaining cells were subdivided into cells located in the inner-most part of the granule cell layer versus cells located in the middle or outer part of the granule cell layer (see Results). This subdivision was performed independently by two investigators blind to the treatment groups and compared afterward. In 96% of the cases the subdivision made by the two investigators was in agreement. In the remaining cases consensus was reached after in-depth investigation of the location.

#### Mossy Fiber Sprouting

Male mice (*n* = 6 per group) were killed between 9 and 12 a.m. under deep pentobarbital anesthesia (200 mg/kg body weight, i.p.) by transcardial perfusion with 0.1% sodium sulfide for 5 min, followed by 4% formaldehyde for 5 min (each in 0.01 phosphate-buffered saline, pH 7.4). Brains were removed from the skull, postfixed at 4°C in 4% formaldehyde/15% sucrose overnight, immersed at 4°C in 30% sucrose in phosphate-buffered saline until they sank and frozen on powered dry ice. Cryostat sections (30 μm) were cut in the coronal plane, mounted on superfrost slides in series of 15 and stored at −80°C until further use. Mossy fibers were stained with Timm histochemistry, according to Danscher (47). Staining was performed in two batches, each containing one slide of every animal to avoid staining-based variation between treatment groups. Sections were developed in the dark for 180 min in a freshly prepared 90/45/15/0.75 (volume/volume) solution of 50% arabic gum, 51% hydroquinone, 25.5% citric acid/23.5% sodium citrate, and 17% silver nitrate. After washing with running tap water, sections were dehydrated using a grading series of ethanol, cleared in xylene and coverslipped using malinol.

Mossy fiber staining in the hippocampal CA3 area and infrapyramidal blade of the DG (the main areas of possible MFS) was scored according to the scoring system described by Holmes et al. (48), ranging from 0 (no staining) to 5 (maximum staining). Eight sections of the septal hippocampus per animal (four sections for both staining batches), within Bregma range −1.46 to −2.80 (coronal), were scored by two independent observers, followed by a consensus meeting. Consensus scores of these eight sections were pooled per animal and the average was used for statistical analysis.

#### Slice Preparation for Electrophysiology

Male mice (*n* = 6–9 per group) were decapitated between 8.15 and 9.15 a.m., a few minutes after taking the animal out of its home cage. Only the first two mice of each litter were used for electrophysiological analysis to avoid confounding effects of a rise in plasma corticosterone due to acute stress. After rapid dissection, the brain was chilled in ice-cold, carbogenated (95% O_2_:5% CO_2_) artificial cerebrospinal fluid (aCSF) containing in mM: NaCl 120, KCl 3.5, MgSO_4_ 5.0, NaH_2_PO_4_ 1.25, CaCl_2_ 0.2, NaHCO_3_ 25.0, and d-glucose 10.0. After removing frontal lobes and cerebellum, 350 μm transversal hippocampal sections were prepared using a vibratome (Leica VT 1000S; Leica Microsystems, Nussloch, Germany). Both hemispheres were separated and all sections were incubated at room temperature in continuously carbogenated aCSF for at least 1 h.

#### Patch-Clamp Recording of Spontaneous Excitatory Postsynaptic Currents (sEPSCs)

Patching of DG neurons was performed using an upright microscope (Nicon Eclipse E600FN) with differential interference contrast and a water immersion objective (×40) to visually identify the cells. The sections were continuously perfused with carbogenated aCSF containing in mM NaCl 120, KCl 3.5, MgSO_4_ 5.0, NaH_2_PO_4_ 1.25, CaCl_2_ 0.2, NaHCO_3_ 25.0, and d-glucose 10 and 20 μM biccuculine to block γ-aminobutyric acid (GABA)_A_-receptor mediated transmission (flow rate 2.0 ml/min, temperature 32°C, pH 7.4). Cell patching was performed as described by Pasricha et al. (49). Briefly, patch electrodes were pulled from a Sutter Instruments Micropipette puller and had a tip resistance of 4–6 MΩ when filled with the pipette (intracellular) solution containing in mM; Cs-methane sulfonate 120, CsCl 17.5, HEPES 10, BAPTA 5, MgATP 2, NaGTP 0.1, pH 7.3 (adjusted with CsOH). An Axopatch 200B amplifier (Axon Instruments, Foster City, CA, USA) was used for whole cell recordings, operating in the voltage-clamp mode. The patch-clamp amplifier was interfaced to a computer *via* a Digidata (type 1322A; Axon Instruments) analog-to-digital converter. Data acquisition was performed with Clampex, version 8.2 (Axon Instruments) at a sampling rate of 50 μs and a 5 kHz Bessel filter. The surface of the section was cleaned to have better vision of the cells in the deeper layers of DG. After establishing a gigaseal, the membrane patch was ruptured and the cell was clamped at a holding potential of −70 mV to allow measurement of currents mediated by the α-amino-3-hydroxy-5-methyl-4-isoxazolepropionic acid (AMPA) receptors, as at this potential, the *N*-methyl-d-aspartic acid or *N*-methyl-d-aspartate receptor is blocked by Mg^2+^. sEPSCs were recorded for 5 min, starting 5–10 min after membrane rupture. After administration of tetrodotoxin (TTX, 0.5 μM, Bioconnect services) for 5 min to block voltage-gated Na channels and consequently also the development of action potentials, miniature EPSCs (mEPSCs) were measured for 5 min. Only one cell was recorded per slice and no more than two recordings were obtained per animal. Only recordings with an uncompensated series resistance of <2.5 times the pipette resistance, <20% variation during the recording period, and with frequencies <2 SD from the mean, were accepted for analysis. For each event, the area under the curve (AUC) and inter-event interval was analyzed using Clampfit version 9.2 (Axon Instruments). The median AUC and inter-event interval per cell were used for statistical analysis.

#### Field Potential Recordings

Sections were transferred to a submersion type recording chamber and continuously perfused with carbogenated aCSF containing in mM: NaCl 124, KCl 2.5, MgSO_4_ 4.0, NaH_2_PO_4_ 1.2, CaCl_2_ 4.0, NaHCO_3_ 26.0, d-glucose 10.0, and 20 μM bicuculline (flow rate 2 ml/min, temperature 32°C, pH 7.4). Field excitatory postsynaptic potentials (fEPSPs) were recorded in the DG, using glass microelectrodes filled with aCSF, positioned in the medial perforant pathway over the suprapyramidal blade of the DG, as confirmed by paired pulse stimulation elicited paired pulse inhibition. Minimum and maximum stimulation intensities were identified. After an incubation period of 20 min, an input–output response curve was generated by gradually increasing the stimulus intensity to define the stimulus intensity that generated the half-maximal response in peak-amplitude; this intensity was used for the remainder of the experiment. To measure paired pulse depression, paired pulses were delivered at an inter stimulus interval of 50, 100, and 200 ms. After 15–20 min of stable baseline recordings, sections were tetanized with 4 trains of 50 pulses of supramaximal intensity at 100 Hz (30 s inter train interval) to induce LTP. Field potentials were recorded for 1 h posttetanus at 30 s intervals. At the end of this period, the post-tetanic paired pulse depression and final input–output curve were determined. LTP was quantified by calculating the ratio between fEPSP slopes recorded post tetanization and pre tetanization. Data were acquired and analyzed with Signal 2.0 software (Cambridge Electronic Design, UK).

### Statistical Analysis

The effect of eFS and injection type on outcome measures was analyzed with a general linear mixed model in a 2 × 3 design, examining the main effects of HT (HT versus NT) and injection type (corticosterone versus vehicle versus none), as well as their interaction. *A priori* we hypothesized that group differences would be caused by HT treatment or injection type. Therefore, not all possible group comparisons were included in statistical analysis. More specifically, in case of a significant main effect of injection type, *post hoc* tests were performed comparing injection types with Bonferroni correction for multiple comparison. Additionally, differences between HT and NT animals were analyzed per injection group with an independent samples *t*-test, Bonferroni corrected for multiple comparison. When data from multiple neurons per animal, or multiple segments per neuron, were analyzed, a linear mixed model was used, including the animal (for dendritic complexity and glutamatergic transmission) or neuron (for spine density) as subject variables. Effects of HT, injection type and their interaction on Sholl distribution were tested with a repeated measure general linear model. Correlation between parameters was assessed with Pearson correlation coefficient. Mean fEPSP slopes pre- and posttetanization were compared with a paired samples *t*-test.

Normality of residues was evaluated with Q–Q plots, variance of residues was evaluated with error plots. Differences were considered statistically significant at *p* < 0.05 (two-tailed) after correction for multiple comparison. Differences that were significant before, but not after correction for multiple comparison (0.05 ≤ *p* < 0.15), were considered trends. Data were analyzed using SPSS 20.0 (SPSS, Inc., Chicago, IL, USA). Unless stated otherwise, data are presented as mean ± SEM.

## Results

### Epileptic Seizures and Neuroendocrine Changes

**Figure 2:**
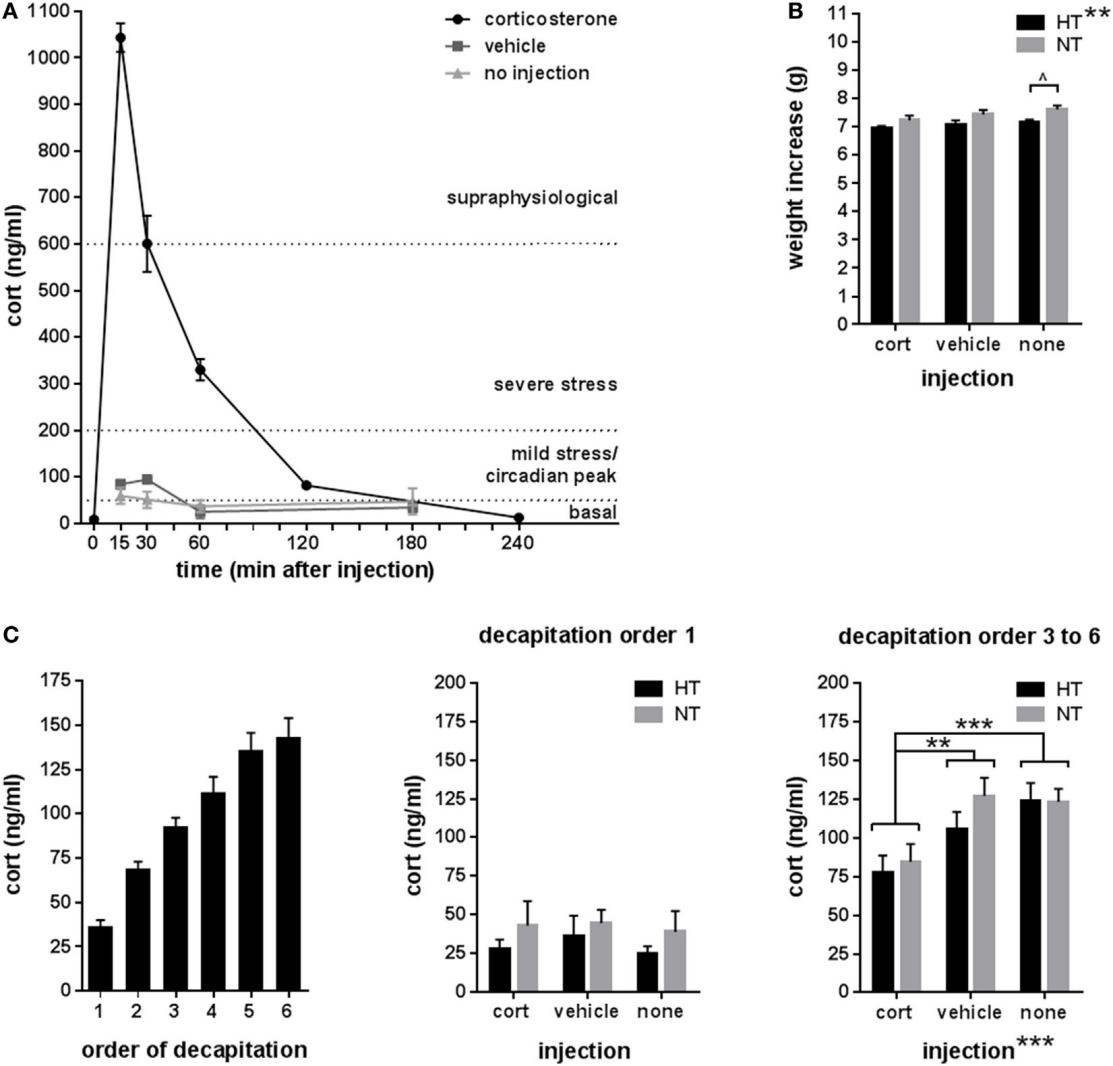
Endocrinology. **(A)** Corticosterone (cort) levels at several time points after single intraperitoneal injection with corticosterone, vehicle, or no injection at 9.00 a.m. Reference corticosterone levels: basal < ±50 ng/ml, circadian peak or mild stress ±50–200 ng/ml, severe stress ±200–600 ng/ml, and supraphysiological > ±600 ng/ml (50–52). **(B)** Weight increase was significantly lower in hyperthermia (HT)- compared to normothermia (NT)-treated animals overall, while analyses per injection type only showed a trend difference in the not injected groups. **(C)** Corticosterone levels after decapitation increased with the order of decapitation within the litter (left panel). In animals decapitated as first of their litter (middle panel), corticosterone levels did not differ between treatment group, while in animals decapitated third to sixth of their litter (right panel), corticosterone levels differed between injection types—with significantly lower levels in the corticosterone injected animals—but not between HT- and NT-treated animals. Data represented as mean ± SEM. ***p < 0.001, **p < 0.01, and ^^^*p* < 0.05 before correction for multiple comparison (trend).

Experimental seizures were elicited in all mice exposed to the HT protocol and the average seizure duration was 26.4 ± 2.0 min. Corticosterone injection resulted in a high peak concentration of corticosterone (1,043.3 ± 28.0 ng/ml at 15 min after injection) with a fast return to baseline in approximately 180 min, while vehicle injection elicited a much smaller (94.6 ± 8.2 ng/ml) and more short-termed increase in corticosterone levels (Figure 2A).

Weight increase between HT/NT treatment and decapitation, a 15 days interval, was significantly lower in HT animals compared to controls [main effect HT, *F*_(1,280)_ = 10.73, *p* = 0.001], but was not influenced by subsequent injections (Figure 2B). No group differences were observed in adrenal and thymus weights (data not shown). Although there were less than 2 min between successive decapitations of animals belonging to the same litter, later decapitation was associated with increased corticosterone levels (Figure 2C, left panel). While no group differences were observed in corticosterone levels in animals decapitated first of their litter (Figure 2C, middle panel), injection type did significantly influence corticosterone levels in animals decapitated as third to sixth of their litter [Figure 2C, right panel, main effect injection, *F*_(2,68)_ = 8.7, *p* < 0.001; corticosterone versus vehicle *p* = 0.009, corticosterone versus no injection *p* < 0.001, vehicle versus no injection *p* = 0.36], indicating that repetitive corticosterone injection indeed affected stress hormone regulation, which confirms successful manipulation.

### Morphology

#### Neurogenesis

**Figure 3:**
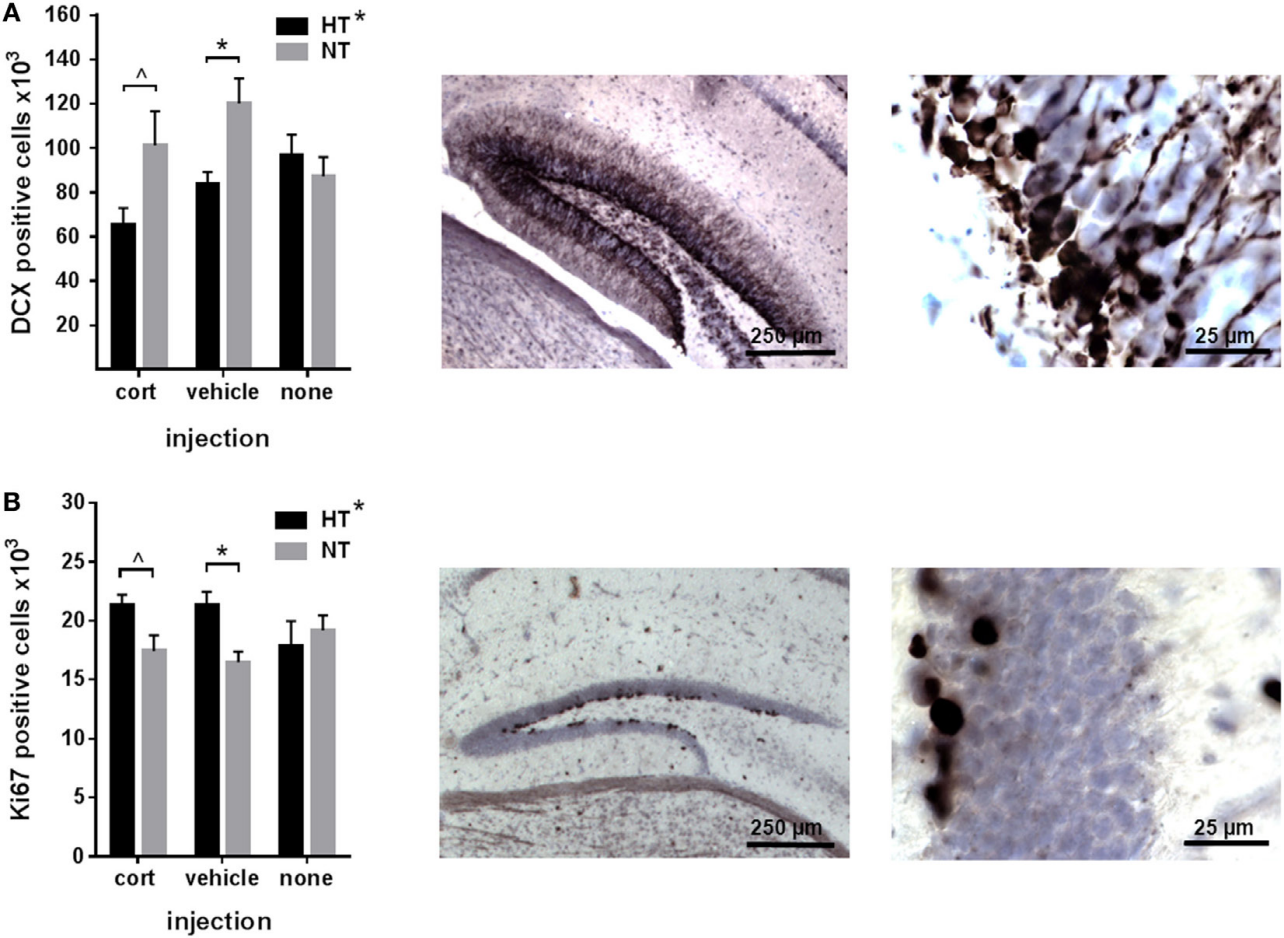
Neurogenesis. **(A)** Left: the number of immature neurons in the dentate gyrus was decreased in male animals exposed to hyperthermia (HT) compared to normothermia (NT) after injection. Middle/right: representative example of DCX staining at lower and higher magnification. **(B)** Left: the number of proliferating cells in the dentate gyrus was increased in males exposed to HT. Middle/right: representative example of Ki67 staining at lower and higher magnification. Data represented as mean ± SEM. *p < 0.05 and ^^^*p* < 0.05 before correction for multiple comparison (trend).

Neurogenesis significantly differed between HT and NT animals. HT was associated with a decrease in the number of immature DCX-positive neurons [main effect HT, *F*_(1,30)_ = 6.21, *p* = 0.019] (Figure 3A), and an increase in the number of proliferating cells in the DG [main effect HT, *F*_(1,30)_ = 4.94, *p* = 0.034] compared to NT (Figure 3B). Accordingly, the number of immature neurons and proliferating cells per animal were negatively correlated (*r* = −0.55, *p* = 0.001). Analyses per injection type revealed that the effects of HT on both DCX- and Ki67-staining were significant in the vehicle injected animals [*F*_(1,10)_ = 12.16, *p* = 0.018, respectively, *F*_(1,10)_ = 15.89, *p* = 0.009], while a similar effect was observed at trend level after corticosterone injection [*F*_(1,10)_ = 5.71, *p* = 0.12, respectively, *F*_(1,10)_ = 6.88, *p* = 0.09]. Non-injected HT and NT groups did not differ at all.

#### Dendritic Morphology

**Figure 4:**
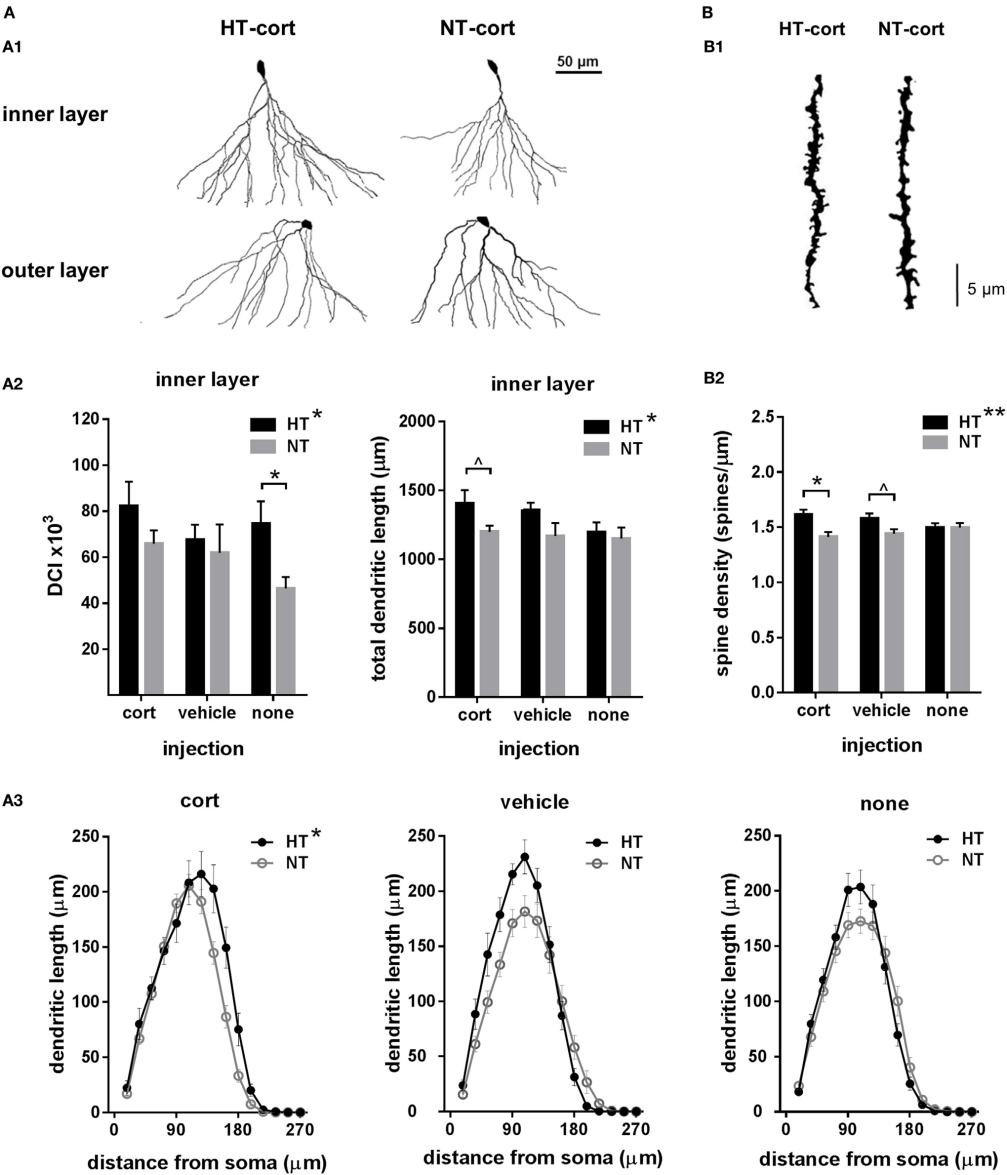
Dendritic morphology. **(A)** Dendritic complexity. (A1) Dendritic complexity differed between dentate granule cells with a cell body located in the granular cell layer or inner part of the molecular layer (inner layer) versus the middle or outer part of the molecular cell layer (outer layer), representative examples of corticosterone (cort) injected animals after hyperthermia (HT) and normothermia (NT). (A2) Left: dendritic complexity index (DCI) in inner layer neurons was increased after HT compared to NT. Right: total dendritic length of inner layer neurons was increased after HT, an effect that increased with injection type. (A3) HT significantly influenced Sholl distribution. **(B)** Spine density. (B1) Representative example of HT-cort and NT-cort animal. (B2) Spine density was significantly higher in animals exposed to HT compared to NT, an effect that increased with injection type. Data represented as mean ± SEM. *p < 0.05 and ^^^*p* < 0.05 before correction for multiple comparison (trend).

To assess the effects of eFS and injection type on dendritic morphology, we evaluated total dendritic length, dendritic reach, dendritic complexity and spine density. As dendritic complexity differed between dentate granule cells with a cell body located in the inner part of the granular cell layer, bordering the subgranular layer (referred to as “inner layer,” *n* = 15–27 per group), versus the middle or outer part of the granule cell layer (referred to as “outer layer,” *n* = 11–25 per group) (Figure 4A, A1), these cells were analyzed separately. In inner layer neurons, dendritic complexity index was increased in HT animals compared to NT controls [main effect HT, *F*_(1,111)_ = 6.23, *p* = 0.014], an effect that was only significant in animals receiving no subsequent injections [*F*_(1,7)_ = 6.64, *p* = 0.04] and was less pronounced after vehicle or corticosterone injection (Figure 4A, A2, left panel). Also the total dendritic length of inner layer neurons was increased in HT animals [main effect HT, *F*_(1,29)_ = 4.47, *p* = 0.04], an effect that aggravated with injection type, although analyses per injection type only revealed a difference in the corticosterone-injected group at trend level (Figure 4A, A2, right panel). No main nor interaction effects were observed on total dendritic reach. Sholl analyses per neuron revealed a main effect of HT [*F*_(1,120)_ = 6.18, *p* = 0.01] and radial distance from the soma [*F*_(3,358)_ = 560.71, *p* < 0.001] on dendrite length, as well as a HT × injection type × radial distance interaction [*F*_(6,358)_ = 3.18, *p* = 0.005] (Figure 4A, A3). In outer layer neurons, no main or interaction effects of HT or injection type were observed on dendritic complexity (data not shown).

Spine density was significantly higher in animals exposed to HT compared to NT [main effect HT, *F*_(1,165)_ = 7.28, *p* = 0.008]. This effect was significant after corticosterone injection [*F*_(1,58)_ = 8.80, *p* = 0.01], at trend level after vehicle injection [*F*_(1,55)_ = 4.67, *p* = 0.11], while it was not observed in animals receiving no injections [*F*_(1,52)_ = 0.00, *p* = 1.00] (Figure 4B, B1,B2).

#### Mossy Fiber Sprouting

**Figure 5:**
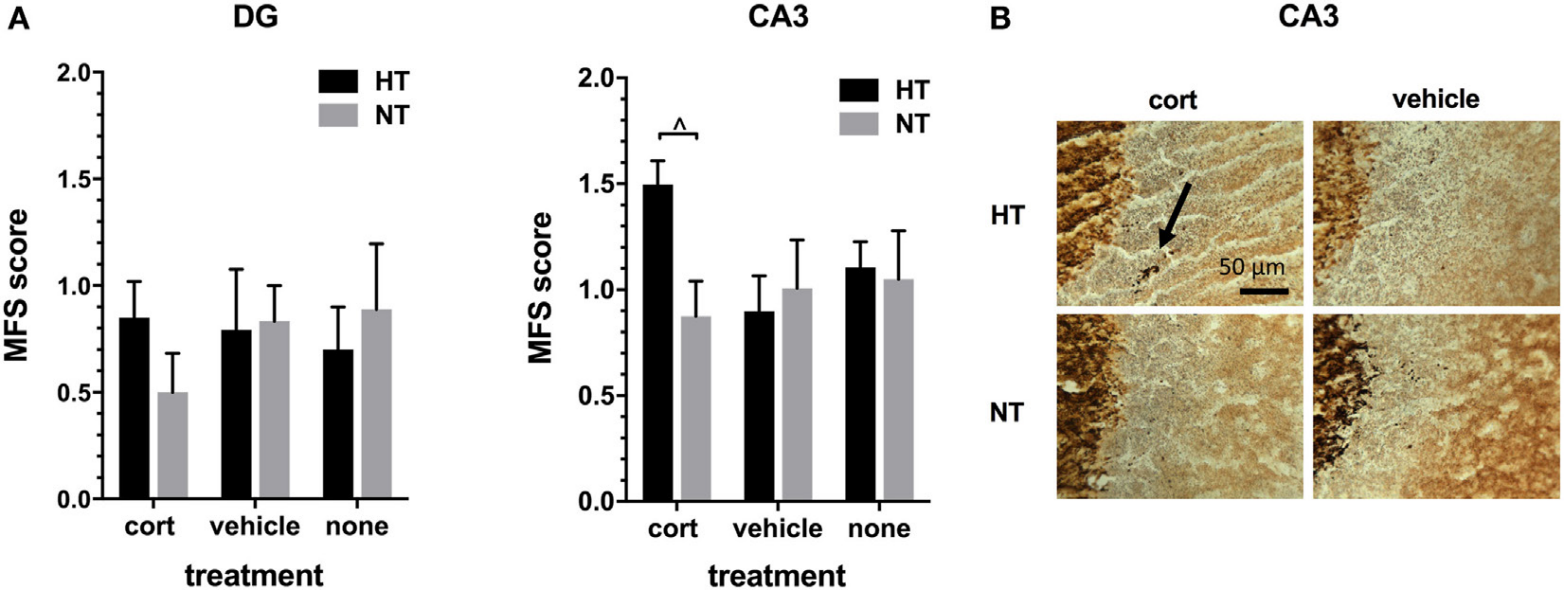
Mossy fiber sprouting (MFS). **(A)** Left: the MFS score in dentate gyrus (DG) did not differ significantly between treatment groups. Right: in the CA3 area of the hippocampus only a trend was seen toward an increase in MFS score after hyperthermia (HT) in animals receiving corticosterone injections. **(B)** Representative example of MFS in the CA3 area in HT and normothermia (NT) animals with corticosterone versus vehicle injection. Arrow: MFS. Data represented as mean ± SEM. ^^^*p* < 0.05 before correction for multiple comparison (trend).

Hippocampal MFS was assessed in the DG and the CA3 area of the hippocampus. As expected, intense Timm staining was observed in the hilus of the DG and in the stratum lucidum of the CA3 area, the main projection sites of mossy fibers. The amount of infrapyramidal Timm staining, characteristic for MFS, was very low in all treatment groups (mean MFS score DG 0.76 ± 0.09, CA3 1.07 ± 0.08, on a scale ranging from 0 to 5). HT or injection type did not significantly affect MFS score in both areas, although there was a trend towards an increased MFS score in the CA3 area after HT in animals receiving corticosterone injections [*F*_(1,10)_ = 7.56, *p* = 0.06, Figure 5].

### Functional Plasticity

The eFS-induced changes in morphology and particularly the increased spine density may affect glutamatergic transmission in the DG, which could be reflected at the level of single cells as well as field potentials. To test this, we examined effects of HT and corticosterone injection on single cell spontaneous synaptic events (which are among other things determined by the number of synaptic contacts) and field excitatory potentials.

#### Single Cell Glutamatergic Transmission

**Figure 6:**
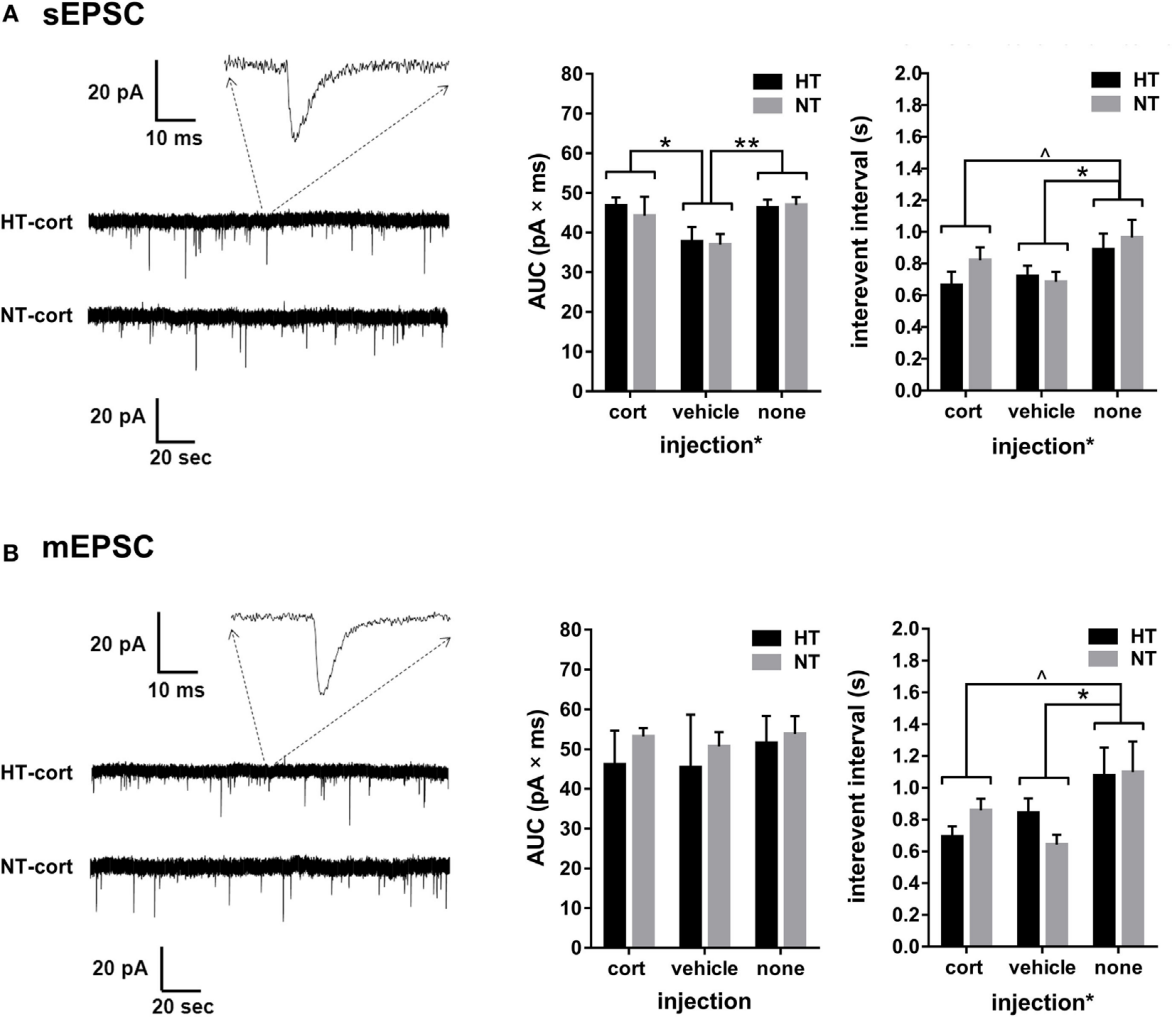
Single cell glutamatergic transmission. **(A)** Left: example showing a spontaneous excitatory postsynaptic current (sEPSC) recording. Middle and right: a main effect of injection type, but not HT, is observed on sEPSC area under the curve (AUC) and interevent interval. **(B)** Left: example showing a miniature excitatory postsynaptic current (mEPSC) recording. Middle: no group differences are observed in mEPSC AUC. Right: a main effect of injection type, but not HT, is observed on mEPSC interevent interval. Data represented as mean ± SEM. **p < 0.01, *p < 0.05, and ^^^*p* < 0.05 before correction for multiple comparison (trend).

Whole-cell voltage-clamp recordings of AMPA receptor mediated EPSCs in the DG were analyzed in 9–11 cells per treatment group. Input resistance and capacitance did not differ between groups (see Table 1). For both sEPSCs and mEPSCs, no HT × injection interaction or main effect of HT was observed. Interestingly, injection type did influence sEPSC and mEPSC properties (Figures 6A,B). A significant main effect of injection type existed on the interval between consecutive events for both sEPSC [main effect injection, *F*_(2,32)_ = 3.48, *p* = 0.03; corticosterone versus vehicle *p* = 0.63; corticosterone versus no injections *p* = 0.06, vehicle versus no injections *p* = 0.01] (Figure 6A, right panel) and mEPSC [main effect injection, *F*_(2,32)_ = 3.95, *p* = 0.03; corticosterone versus vehicle *p* = 0.65; corticosterone versus no injections *p* = 0.07, vehicle versus no injections *p* = 0.01] (Figure 6B, right panel), with a lower inter-event interval in the vehicle injection group compared to the non-injection group; a similar difference, although only at trend level, was observed for the corticosterone-injection versus non-injection groups. Also the AUC of the sEPSCs was influenced by injection type [*F*_(2,36)_ = 4.39, *p* = 0.02; corticosterone versus vehicle *p* = 0.04, vehicle versus no injections *p* = 0.004, corticosterone versus no injections not significant] with a smaller AUC after vehicle injection compared to the other groups (Figure 6A, middle panel), while the AUC of mEPSC did not significantly differ between groups (Figure 6B, middle panel).

**Table 1:** Baseline measurements of single cell glutamatergic transmission and synaptic plasticity.

Treatment group	Single cell transmission		Synaptic plasticity	
	Input resistance (mΩ)	Capacitance (pF)	Baseline slope (V/s)	Stimulation intensity (mA)
HT-cort	336 ± 49	9.8 ± 0.7	−0.13 ± 0.02	0.63 ± 0.05
NT-cort	338 ± 38	9.0 ± 0.8	−0.20 ± 0.02	0.71 ± 0.03
HT-vehicle	303 ± 33	9.0 ± 0.7	−0.19 ± 0.02	0.66 ± 0.02
NT-vehicle	320 ± 38	9.2 ± 0.7	−0.14 ± 0.03	0.77 ± 0.08
HT-none	285 ± 35	9.4 ± 0.8	−0.22 ± 0.02	0.80 ± 0.12
NT-none	302 ± 37	9.6 ± 1.1	−0.22 ± 0.04	0.84 ± 0.14

*Baseline measurements of functional plasticity were not significantly influenced by the HT × injection type interaction, HT, or injection type. Data represented as mean ± SEM*.

#### Synaptic Plasticity

**Figure 7:**
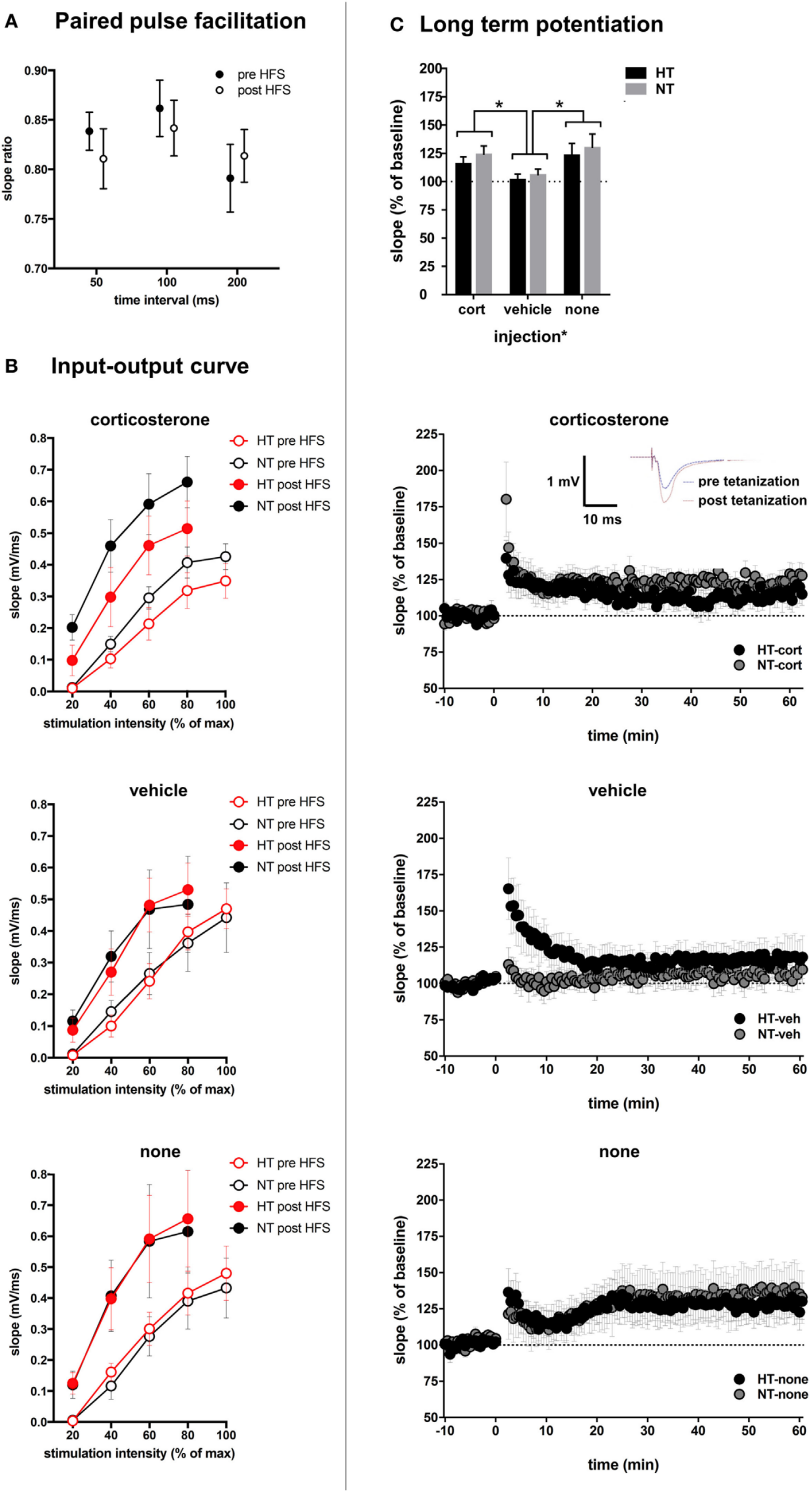
Synaptic plasticity. **(A)** Paired pulse facilitation before and after high-frequency stimulation (HFS). **(B)** The input–output curve is increased after HFS, but did not differ significantly between treatment groups. **(C)** Top: injection type, but not HT, influenced field-evoked postsynaptic potentials (fEPSP). Upper middle: LTP recordings of fEPSP in hyperthermia (HT) vs. normothermia (NT) animals after repetitive corticosterone injection (tetanization started at *t* = 0). Inset: example showing increased slope and amplitude of fEPSP after tetanization. Lower middle: fEPSP in HT vs. NT animals after repetitive vehicle injection. Bottom: fEPSP in HT vs. NT animals without subsequent injections. Data represented as mean ± SEM, *p < 0.05.

Changes in glutamatergic transmission at the single cell level may alter circuit properties and the ability to induce synaptic plasticity. This was tested with field potential recording and application of high-frequency stimulation. Treatment groups did not differ with respect to baseline slopes or stimulation intensity necessary to produce the half-maximal fEPSP, suggesting that HT and injection type did not influence baseline-evoked transmission (see Table 1). No significant differences were observed in paired pulse facilitation before and after high-frequency stimulation (Figure 7A). The input-output curve was increased after high frequency, and did not differ significantly between treatment groups (Figure 7B). A significant increase in fEPSP slope post tetanization (LTP) was elicited in 71% of all animals (38–88% per group). Synaptic plasticity at half-maximal or maximal stimulation was not significantly influenced by the HT × injection interaction or by HT, but only by injection type [main effect injection, *F*_(2,38)_ = 4.20, *p* = 0.02; corticosterone versus vehicle *p* = 0.02, corticosterone versus no injections *p* = 0.44, vehicle versus no injections *p* = 0.01, other group comparisons not significant] (Figure 7C). At maximum stimulation intensities, no group differences were observed.

## Discussion

To increase understanding of the effects of stress and stress hormones on early-life epileptogenesis, we induced epileptogenesis in young mice using the HT-induced eFS model and subsequently exposed them to (1) corticosterone, (2) vehicle injection (a mild stressor), or (3) no injections, and evaluated morphological and functional parameters in the DG. In this latent phase of early epileptogenesis, few effects of eFS were observed in animals that were not subsequently injected. However, in mice that received repetitive corticosterone or vehicle injection (mild stress), eFS induced epileptogenesis was associated with changes in DG morphology, namely a lower number of immature cells, an increased cell proliferation and an increased dendritic length and spine density. These morphological changes associated with epileptogenesis did not translate into differences in glutamatergic functional plasticity.

### Experimental Model

The eFS model is a model of early-life prolonged febrile seizures with close resemblance to the human situation (30, 31). The relatively long-lasting latent phase of epileptogenesis in this model provides the unique opportunity to study epileptogenesis in the absence of spontaneous seizures and relatively apart from the damage and compensatory mechanisms that may arise due to recurrent electrographical or clinical seizure activity. However, non-epileptogenic effects of the initial eFS cannot be fully excluded. As we were interested in epileptogenic changes, outcome measures were assessed before the onset of spontaneous seizures. Based on earlier literature, a subset of animals exposed to eFS is expected not to develop epilepsy (26, 35, 36). However, the normal distribution of our outcome measures suggests a gradual distribution of epileptogenesis among animals. Further studies measuring long-term (seizure) outcome are required to determine whether the morphological and functional measures assessed are stable during the course of epileptogenesis, as well as their relevance for epilepsy outcome.

We hypothesized the mild epileptogenic changes in the latent phase after eFS to be aggravated by stress (hormone) exposure. As different stressors might differentially affect seizure-susceptibility [reviewed in Ref. (2, 23, 53)], we decided to use a clean design of injections with corticosterone—the end product of the stress response, exerting large effects on brain structure and function, including neuronal excitability (22)-, and control groups of mild injection stress and undisturbed animals. Stress paradigms in rodents are usually associated with a reduction in bodyweight and effects on adrenals and thymus. In our experiment, we did not observe such changes, possibly due to the mild nature of the injection stress. However, the reduced corticosterone levels in corticosterone-injected animals that were decapitated after prior handling of littermates, which can be considered an acute stressor, indicate that corticosterone injections *did* downregulate responsiveness of the hypothalamic–pituitary–adrenal axis. The effects of these supraphysiological doses of corticosterone were smaller than expected, which might relate to the young age of the animals, just after the stress hyporesponsive period. Also, the transient increases in corticosterone levels in the morning may not have interfered with the ultradian pulsatility of endogenous hormone levels (54), benefiting its comparability to real life stress exposure.

### Effects of Corticosteroids on Structural Plasticity during Epileptogenesis

In animals that were *not* subsequently injected, HT only affected dendritic complexity, but none of the other outcome measures. The negative results during this early phase of epileptogenesis are in line with previous studies that also reported no differences in MFS (24, 27) or DG neurogenesis (24) around this age. Similarly, in non-injected mice we observed no effects of HT on DG spine density.

Differences between animals exposed to HT and NT only became manifest after repetitive corticosterone or vehicle injections. The increase of HT-associated changes after stress (hormone) exposure is in line with the vast amount of previous studies reporting stress exposure *before seizure induction* to increase seizure-susceptibility and seizure-severity [reviewed in Ref. (55)]. The effects of HT combined with corticosteroids or mild stress on proliferation are similar to those reported at a later stage of epileptogenesis after HT only (25, 26), suggesting that corticosterone and stress *accelerate* epileptogenesis-related structural plasticity. We observed an inverse relation between the number of proliferating cells and the amount of immature neurons, suggesting that the increased proliferation mainly occurs in non-neuronal (e.g., glial) cells, consistent with the gliosis prominent in many epilepsy models (56, 57).

Dendritic length, complexity and spine density are generally reported to decrease after seizures (58–63), which is considered a compensatory mechanism in response to the excess of excitatory input. Dendritic complexity was previously shown to be increased after eFS (28). The enhanced dendritic length and spine density that we found during the latent phase of early epileptogenesis in combination with corticosterone exposure and mild stress is therefore likely to be part of the epileptogenic process.

Clearly, the parameters that we investigated might not only be altered by epileptogenesis but also by corticosterone and stress themselves. This is shown for instance by the increased number of DCX^+^ cells in the vehicle injected compared to non-injected NT group, that is consistent with the literature (21, 64).

### Effects of Corticosteroids on Functional Plasticity during Epileptogenesis

In non-injected animals, glutamatergic transmission and LTP were unaffected by HT. The latter is somewhat surprising, as around this age HT was shown to affect GABAergic transmission in DG (65) and both glutamateric and GABA-ergic transmission in CA1 (27, 66–69). Since we did not record GABAergic signals, we cannot exclude that in our model GABAergic transmission in the DG might have been altered.

In view of the increased dendritic length and spine density observed after HT when followed by corticosterone (and to a lesser degree vehicle) injection, the latent phase of early epileptogenesis may be accompanied by an expanded postsynaptic “potential” for synaptic transfer of signals. We tested whether this translated to the functional level; i.e., a higher spine density may result in increased mEPSC frequency and this, in turn, may enhance the ability to induce LTP (70). However, this appeared not to be the case. Rather than HT, the condition of mild stress related to vehicle injection resulted in a higher frequency of both sEPSCs and mEPSCs, and a smaller AUC of sEPSCs; the latter could explain the reduced ability to induce LTP in these groups. The discrepancy between structural changes in dendrites and spines versus sEPSC frequency is unexpected, but emphasizes that presynaptic changes, e.g., after mild injection stress, are important for the overall outcome in functional terms. Presynaptic effects of stress or corticosterone are indeed well documented (71, 72), also in the mouse DG (49), although these were never investigated with this particular paradigm and at this age. The data furthermore illustrate that the functional effect of mild injection stress cannot be extrapolated to effects of a high dose of corticosterone. This may relate to a bell-shaped dose-dependency for corticosterone, as indeed often observed (73, 74) or, importantly, the fact that mild (injection) stress causes the release of several stress-related hormones in addition to corticosterone.

### Potential Implications for Epileptogenesis

The increase in morphological differences after HT when followed by corticosteroid and mild stress exposure suggests that stress (hormones) aggravates or accelerates epileptogenesis. Epileptogenesis, i.e., the neurobiological processes leading to epilepsy, is not limited to the time before the onset of spontaneous seizures, but continues during the course of epilepsy, contributing to the progression of the disease (75, 76). As none of the currently available anti-epileptic drugs can favorably modify the disease process, prevention or reduction of epileptogenesis remains one of the main challenges in the field of epilepsy (77–79) and could be beneficial for all patients, irrespective of the underlying pathology. Children with complex febrile seizures are of special interest, as they have a high risk to develop epilepsy—ranging from 21% after prolonged febrile seizures (80) to as much as 49% after prolonged seizures with focal semiology that reoccur within 24 h (81)—that is already identified at the very beginning of epileptogenesis, making them very suitable for early intervention. Large population-based studies, prospectively following children after complex febrile seizures and systematically documenting stress exposure, as well as epilepsy outcome, can provide more insight into the effects of stress on epileptogenesis in this human population.

In conclusion, our results suggest that stress and stress hormones modulate epileptogenesis, indicating that stress reduction strategies and possibly even medication targeting the stress system may have a potential role in reducing epileptogenesis. As studying epileptogenesis apart from seizure frequency is difficult, especially in humans, animal studies could provide valuable information on the effects of stress reduction on epileptogenesis, for example, by studying effects of an enriched environment during the latent phase of epileptogenesis on seizure outcome.

## Ethics Statement

All experimental procedures were performed according to the institutional guidelines of the University Medical Center Utrecht and approved by the committee on ethical considerations in animal experiments of Utrecht University (DEC Utrecht, permit number 2012.I.03.047).

## Author Contributions

JC, KPJB, PG and MJ contributed conception and design of the study. JC, EH, KB, GR, ST, EU, and GM contributed to data acquisition. JC organized the database, performed the statistical analysis, and wrote the first draft of the manuscript. JC, KB, GR, PL, PG, and MJ contributed to data interpretation. All authors contributed to manuscript revision, read, and approved the submitted version.

## Conflict of Interest Statement

The authors declare that the research was conducted in the absence of any commercial or financial relationships that could be construed as a potential conflict of interest.

## Abbreviations

DG, dentate gyrus; eFS, experimental febrile seizures; HT, hyperthermia; NT, normothermia.
